# Understanding the influence of suicide bereavement on the cognitive availability of suicide: Qualitative interview study of UK adults

**DOI:** 10.1111/sltb.13134

**Published:** 2024-11-04

**Authors:** Poppy Jones, Katie E. Quayle, Sunjeev K. Kamboj, Martina Di Simplicio, Alexandra Pitman

**Affiliations:** ^1^ Research Department of Clinical, Educational and Health Psychology University College London (UCL) London UK; ^2^ North London NHS Foundation Trust London UK; ^3^ Imperial College London London UK; ^4^ University College London, Division of Psychiatry London UK

**Keywords:** bereavement, suicidal ideation, suicide, traumatic loss

## Abstract

**Background:**

People bereaved by suicide are at increased risk of suicide. Potential explanations include changes in the cognitive availability of suicide after suicide bereavement, but this has been under‐investigated. This study aimed to investigate how suicide bereavement influences thoughts about suicide, including methods considered.

**Method:**

We interviewed 20 UK‐based adultswho reported having been preoccupied by the suicide of a close contact, analyzing qualitative data using reflexive thematic analysis.

**Results:**

We identified four main themes: divergent changes in views about suicide as an option; impact of the method used on consideration of own potential method of suicide (including an aversion to the same method); experience of suicidal ideation as a means of understanding the deceased's state of mind; and thoughts related to reunion with the deceased.

**Conclusions:**

Our findings suggest that the trauma of exposure to a close contact's suicide can modify the cognitive availability of suicide in divergent ways, including suicide being perceived as a more or less acceptable option, and a tension between the two. These insights assist clinicians in sensitive exploration of suicide bereavement and in risk mitigation. They suggest revisions to existing models of cognitive availability and the potential for psychological interventions that modify the cognitive availability of suicide.

## INTRODUCTION

Globally, approximately 700,000 people die by suicide annually (World Health Organization, 2021), with estimates of between six (Clark & Goldney, 2000) and 135 (Cerel et al., 2019) people emotionally affected by each suicide loss. Suicide bereavement is associated with a range of adverse mental health outcomes, including suicide attempt and suicide (Erlangsen et al., 2017; Pitman et al., 2014). Consequently, the majority of suicide national prevention strategies recommend provision of support following suicide bereavement (Schlichthorst et al., 2022). However, efforts to prevent suicide in this group are hindered by a lack of understanding of the mechanisms of suicide risk after suicide loss.

Factors such as heritability of suicidality, chronic stress, disrupted attachments, and social modeling may influence suicide risk following suicide bereavement (Hua et al., 2020). Many theoretical models also point to cognitive processing of others' suicidal behavior as a factor influencing own suicide risk, warranting empirical investigation. The Interpersonal Theory of Suicide defines acquired capability for suicide as a heightened degree of fearlessness and elevated physical pain tolerance such that the actions and ideas involved in suicide are no longer alarming, acting on suicidal thoughts is facilitated, and evolutionary programming for self‐preservation is over‐ridden (Joiner, 2005; Van Orden et al., 2010). A reduced fear of death might be engendered through experiences of others' deaths, knowledge of methods used, and own self‐harm or suicide attempt (O'Connor & Kirtley, 2018). Acquired capability for suicide may therefore be one mechanism by which suicidal thoughts and behavior might emerge following suicide bereavement (Erlangsen et al., 2017; Pitman et al., 2016; Tidemalm et al., 2011).

A related construct, cognitive availability, describes an individual's awareness of suicide as an option and knowledge about possible means of suicide (Florentine & Crane, 2010) or their ‘psychological closeness’ to a suicide method (Rogers et al., 2019). This includes knowledge and beliefs about accessibility, painfulness, lethality, and technical aspects of a specific method (Barber & Miller, 2014; Florentine & Crane, 2010; Sarchiapone et al., 2011). Such familiarity can arise directly from personal experience of one's own or others' suicidal behavior (Biddle et al., 2012) and/or indirectly through media depictions of suicidal behavior (Niederkrotenthaler et al., 2020; Pirkis & Blood, 2001; Posselt et al., 2021). Whilst there is epidemiological evidence that risk of suicide attempt is increased after the suicide of a relative or friend (Erlangsen et al., 2017; Pitman et al., 2016; Tidemalm et al., 2011), after exposure to the suicide of someone in one's social circle (Hill et al., 2020) and after irresponsible media reporting (Niederkrotenthaler et al., 2020) or depiction of suicide (Bridge et al., 2020), little research has explored underlying cognitive processes. Previous qualitative studies with suicide‐bereaved adults suggest that for some people a close contact's suicide can normalize suicide as a form of problem‐solving, whilst for others it can create a determination to avoid suicide and its devastating impact (Bell et al., 2015; Grimmond et al., 2019; Miklin et al., 2019; Pitman et al., 2017). These findings suggest that personal experiences can influence one component of cognitive availability, namely an individual's awareness of suicide as an option, which in turn may contribute to acquired capability for suicide. However, empirical work is required to explore how direct exposure to suicide influences a bereaved person's thoughts about their own suicide and specific methods. This study aimed to address this evidence gap by investigating the perspectives of suicide‐bereaved adults in relation to their own thoughts about suicide after the suicide of a close contact.

## MATERIALS AND METHODS

### Study design and participants

We conducted a qualitative interview study in a community sample of suicide‐bereaved adults, using reflexive thematic analysis to explore attitudes towards suicide following the suicide of a close contact. In one‐to‐one interviews we probed bereaved individuals' processing of the deceased's suicidal behavior and of the specific suicide method used, in order to explore and characterize the ways in which they had thought about suicide after direct experience of suicide loss.

We recruited UK‐based suicide‐bereaved adults using social media advertisements and the email distribution list of the national organization representing a network of UK suicide bereavement support organizations (Support After Suicide Partnership). The advertisement invited suicide‐bereaved adults to take part in an interview study to explore their “experiences of thoughts and images following a bereavement by suicide.” This invitation recruited for two linked studies: one investigating the experience of intrusive mental imagery after suicide bereavement (Quayle et al., 2023) and the current study investigating the perspectives of those who were preoccupied by the suicide of a close contact. A weblink guided prospective participants for both studies to the project webpage, which included the participant information sheet, an online screening questionnaire, and a list of support sources. Potential participants were asked to complete the screening questionnaire to establish basic demographic information and brief details of thoughts related to their close contact's suicide. This and a telephone screening interview helped ascertain eligibility for either study, to help with purposive sampling, and to assess risk. One participant contributed a separate interview for each study.

Inclusion criteria for the current study were: English‐speaking; aged ≥18 years (bereaved at any age); bereavement by suicide of a close contact (a relative or friend who mattered to the participant and from whom they obtained emotional or practical support); reported being preoccupied by the suicide of a close contact.

Exclusion criteria were: recent suicide bereavement (≤6 months); recent suicide attempt (≤6 months); and apparent cognitive impairment. We did not screen for pre‐existing mental health disorders as this was not an exclusion criterion.

Of 85 people volunteering for an interview for either study, we excluded 4 who resided outside the UK or provided insufficient contact details. We purposively sampled 20 individuals who met criteria for the current study, aiming to achieve as much diversity as possible based on age, gender, ethnicity, kinship to the deceased, and time since bereavement. As coding proceeded in parallel to interviews (see below) our team discussions included consideration of the point at which to stop sampling, based on our appraisal of the adequacy (richness and complexity) of the data for addressing the research question, whilst acknowledging the debate about the concept of saturation (Braun & Clarke, 2021b).

### Procedure

We developed an interview topic guide for this study (Appendix S1) in collaboration with lived experience experts from the Support After Suicide Partnership. Questions prompted each participant to describe the lead‐up to the suicide, their thoughts and experiences following the bereavement, and any impact of the bereavement on thoughts about their own suicide, and ways of coping. The topic guide was sufficiently flexible to allow for exploration of unanticipated aspects of participants' experiences and did not require iterative amendment over the course of interviews.

All interviews for this study were conducted between 08/07/20 and 13/11/20 by one researcher (first author) who was a trainee clinical psychologist. Interviews were conducted using an online video platform, lasting between 54 and 90 min, and were audio recorded. The interviewer was clear about the right to pause at any point or to end the interview. At the end of each interview, the researcher reassessed risk and emailed the participant an electronic copy of Help is at Hand, a UK suicide bereavement support guide (Public Health England, National Suicide Prevention Alliance, 2015). The researcher also sent a follow‐up email one week later to check on welfare.

Participants were provided with the opportunity to review a copy of the transcript for accuracy. Seven participants requested a copy and two responded with comments or amendments.

### Ethics

The study was approved by the UCL Research Ethics Committee (16,587/001). All participants provided written informed consent. At the screening stage we used an online questionnaire to collect information on participants' location and GP contact details, explaining that this formed part of our risk protocol (Appendix S2) should we need to contact the GP about risk concerns.

### Data analysis

We analyzed data using the approach of reflexive thematic analysis (Braun & Clarke, 2006, 2019, 2021a) to identify themes representing patterns of shared meaning in relation to thoughts about suicide after the suicide of a close contact. We positioned this within a social constructivist paradigm (Galbin, 2014), recognizing that an individual's interpretation of their experiences of suicide will be influenced by societal and cultural norms about suicide (Dempsey et al., 2023). Our team was composed of clinical academics with research training in social psychiatry, psychiatric epidemiology, imagery phenomenology, cognitive psychology and cognitive neuroscience. We were mindful of the influence of the perspectives we brought to the interviews and analysis, particularly our theoretical assumptions based on existing theories of suicidal behavior. As part of this process, midway through the course of the interviews, the interviewers for the two linked studies engaged in a bracketing interview with each other in order to facilitate reflection on their own position in the research project and address any biases (Creswell & Miller, 2000).

In the first of the six phases of reflexive thematic analysis the same interviewer manually transcribed audio recordings verbatim immediately afterwards to enhance familiarization. Familiarization among the wider analytic team was achieved through (i) the interviewer debriefing to the second author (also involved in coding) after each interview and (ii) all transcripts being read by the senior author. Stage two (coding) occurred in parallel with interviews and team coding discussions. Data management was facilitated using NVivo (QSR International, 2018). Initially two transcripts were coded independently by two researchers (first and second authors), both trainee clinical psychologists, comparing codes to agree an initial coding framework and checking codes against reflective journals and outliers. The coding framework was reviewed iteratively as coding proceeded in discussion with the wider research team. Stage three involved generating initial themes from codes, again reviewed with the wider research team. Stage four involved further development and reviewing of themes, continuing to discuss coding and interpretation of results among the team to explore differences in interpretation of narratives, improve the consistency of coding, and reduce the influence of personal reflexivity. In stage five we refined the thematic framework and reviewed the names of themes. Finally, in stage six we wrote the report following COREQ guidelines (Tong et al., 2007), illustrating themes with quotes whilst revising the thematic framework and names of themes. We decided to communicate the patterning of themes visually to convey the conflicting positions that some interviewees described.

## RESULTS

### Sample characteristics

Our sample of 20 adults was predominantly female (80%) and White (80%) with a mean age of 45 years (Table 1). The majority (18/20) described a history of suicide of one close contact, but two participants had each been bereaved by the suicide of two close contacts.

**TABLE 1:** Characteristics of study participants (*n* = 20).

	(*n* = 20)	
Characteristics of the bereaved	*n*	(%)
Gender		
Female	16	(80)
Male	4	(20)
Participant age (Years)		
Range	23–64	
Mean (*SD*)	44.75 (12.48)	
Ethnicity	*n*	(%)
White British	16	(80)
White British/White Irish	1	(5)
Other white	1	(5)
Mixed race^a^	2	(10)
Country of residence	*n*	(%)
England	15	(75)
Scotland	2	(10)
Wales	3	(15)
Northern Ireland	0	0 (0)
Age of deceased (Years)		
Range	13–70	
Mean (SD)	34.59 (*15.54*)	
Time since bereavement (Years)		
Range	1.36–24	
Mean (SD)	5.71 (7.30)	
Characteristics of the deceased^b^ ^,^ ^c^	(*n* = 22)	
Gender of the deceased		
Male	16	(73)
Female	6	(27)
Kinship to the deceased	(*n* = 22)	
Blood Relative	15	(68)
Non‐blood relative	7	(32)
Relationship to the deceased^b^	(*n* = 22)	
Mother	2	(9)
Father	1	(5)
Brother	4	(18)
Sister	1	(5)
Daughter	3	(14)
Son	3	(14)
Husband	2	(9)
Partner	2	(9)
Ex‐Partner	1	(5)
Cousin	1	(5)
Friend	2	(9)
Cause of death^b^	(*n* = 22)	
Hanging or strangulation	15	(68)
Drowning	3	(14)
Jumping or lying in front of a moving object	2	(9)
Fall	1	(5)
Self‐poisoning	1	(5)

^a^ Mixed race ethnicities include White and Black African and White and Asian;

^b^ Sum of percentages for characteristics of the deceased and cause of death do not equal 100% due to rounding of integers.

^c^ *n* = 18 of participants reported the suicide loss of one close contact; *n* = 2 participants each reported the suicide loss of two close contacts.

### Themes

We identified four main themes capturing the nature of reflections about own suicide following suicide bereavement: (1) divergent changes in views about suicide as an option; (2) impact of the method of suicide on consideration of own potential methods of suicide; (3) experience of suicidal ideation as a means of understanding the deceased's state of mind; and (4) thoughts related to reunion with the deceased. We present these below together with sub‐themes and illustrative quotes. Table 2 represents the distribution of sub‐themes across participants.

**TABLE 2:** Table of themes by participant.

	P1	P2	P3	P4	P5	P6	P7	P8	P9	P10	P11	P12	P13	P14	P15	P16	P17	P18	P19	P20
1. Divergent changes in views about suicide as an option	◆	◆	◆	◆	◆	◆	◆	◆	◆	◆	◆	◆	◆	◆	◆	◆	◆			◆
1.1. Suicide becomes an option		▲	▲	▲	▲		▲		▲	▲	▲				▲	▲	▲			▲
1.1.1. Suicide becomes a legitimate option		△	△	△					△	△	△				△	△	△			△
1.1.2. Normalized dying by suicide			△							△					△					△
1.1.3. The comfort of having suicide as an option			△	△	△				△	△	△						△			△
1.1.4. Reduced fear of death							△			△	△				△	△				
1.2. Deterred from suicide being an option	▲			▲	▲	▲		▲	▲		▲	▲	▲	▲			▲			▲
1.2.1. Not wanting to inflict the same suffering on others	△			△	△	△		△	△		△	△	△	△			△			△
1.2.2. Suicide being a permanent solution to a temporary problem	△												△	△						
1.3. Conflicting views on suicide as a potential option				▲	▲				▲		▲						▲			▲
2. Impact of the method of suicide on consideration of own potential methods of suicide			◆	◆			◆		◆	◆					◆		◆		◆	◆
2.1. Aversion to the same method			▲	▲			▲		▲	▲					▲		▲		▲	▲
2.2. A desire to protect others from the trauma of discovery				▲					▲	▲										▲
2.3. Concerns around being found alive and wanting to guarantee own death									▲	▲					▲		▲			▲
3. Experience of suicidal ideation as a means of understanding the deceased's state of mind			◆				◆		◆	◆					◆		◆	◆	◆	◆
4. Thoughts related to reunion with the deceased			◆							◆					◆	◆	◆	◆		

#### Theme 1: Divergent changes in views about suicide as an option

Eighteen participants described how the suicide of their close contact had impacted upon their own thoughts about suicide, changing them so that they viewed suicide as a more feasible consideration for them, became determined to avoid it, or felt conflicted between the two. Their responses indicated that having not apparently reflected much about suicide previously, their experience had brought it to the forefront. Many had started to consider carefully whether this was an “option” for them; a term featuring across numerous accounts.

##### Sub‐theme 1.1: Suicide becomes an option

Twelve participants described how the experience of suicide loss had engendered a sense of suicide being a more “viable” or “valid” option, providing varied rationales.

###### Suicide becomes a legitimate option

Ten participants explained that suicide now seemed a more “viable option”, viewing it as a “back‐up” plan when faced with adversity.

*“If it's so unbearable*…*I can always do that. It's almost your get*‐*out card*…*which you would never have considered before, but now, well there's always that.”* (Female, 60s, bereaved by the suicide of her ex‐partner).

Some described the way the deceased's act seemed to have legitimized the option of suicide when feeling trapped.

“*I didn't ever think about suicide like I do now as an option because*, *I dunno*, *I had never been in that place*, *whereas after* [*she*] *died*, *I think it becomes a reality*” (Female, 20s, bereaved by the suicide of her sister).

“*It was almost like*… *if she's done it, then what's to stop me doing it*?…*that seemed like a viable option to me*.” (Female, 40s, bereaved by the suicide of her adolescent daughter).

###### Normalized dying by suicide

Four participants felt that the suicide had normalized suicide as something that people ‘like them’ might do when facing adversity, previously having felt quite distanced from the idea of attempting suicide.

*“I think because [she] had done it, it almost normalized it for me.”* (Female, 40s, bereaved by the suicide of her adolescent daughter).

*“*…*it becomes a reality, it becomes something that people do.”* (Female, 20s, bereaved by the suicide of her sister).

Proving a perspective on pre‐bereavement attitudes to suicide, one participant explained:

*“it almost just felt like it was something that, you know, famous people died of drug overdoses, or, you know, it felt like something very not real to me, and I guess, now it does feel very real to me*…*it can happen and it can be done by normal people.”* (Female, 20s, bereaved by the suicide of her father).

###### The comfort of having suicide as an option

Eight participants found it comforting to think that suicide was an escape option for them when faced with hardship, even if uncertain about whether they would act on this.

*“*…*I find it quite comforting, that you know, there's an option there to stop the pain, as it were.”* (Male, 40s, bereaved by the suicide of his friend in young adulthood).

*“*…*it feels like a comfort to know that there's that fallback*…*I've already decided like if things get too bad, I now know there's an answer”* (Female, 40s, bereaved by the suicide of her brother and her cousin).

These accounts suggested that the sense of comfort provided by this escape route reduced anxiety about the future.

###### Reduced fear of death

Five participants described a diminishment of their fear of death and an awareness that this might reduce an aversion to attempting suicide if things became difficult. This was a frightening combination for some but others described this erosion of fear in a dispassionate way.

*“*…*one of the things that has persisted is it's made me a little bit less scared of death*. (Female, 40s, bereaved by the suicide of her adolescent daughter).”

*“As in the fear of death is not that big anymore.”* (Male, 50s, bereaved by the suicide of his adolescent son).

For some, the fearlessness was so striking they expressed surprise at still being alive.

“*I dunno how I managed to stop myself from doing it, because the urge was so high.”* (Female, 20s, bereaved by the suicide of her boyfriend).

##### Sub‐theme 1.2: Deterred from suicide being an option

Twelve participants reported that the experience of suicide bereavement had created a resolve to avoid attempting suicide. They provided a range of rationales, presented below, reflecting prolonged rumination over ‘*a pros and cons list*’ relating to the option of suicide.

###### Not wanting to inflict the same suffering on others

All twelve participants described in some detail the emotional impact of the suicide on themselves, other close contacts, and those involved in discovering the body. They all acknowledged that the potential to inflict similar horror on others was a deterrent against choosing suicide, despite offering them a chance of escape.

*“I think it leaves [a] horrible legacy for the people left behind that's difficult to come to terms with. Having witnessed this, I wouldn't want to put them through it.”* (Male, 60s, bereaved by the suicide of his daughter).

*“*…*I can't do it because I've seen what it's done to everyone else.”* (Female, 20s, bereaved by the suicide of her mother).

*“I know I couldn't do it. The mess that he left behind, the trauma that it caused, the distress, the damaged lives. Not a chance would I want to be responsible for that.”* (Female, 50s, bereaved by the suicide of her brother).

For those who had discovered the body or gleaned what this experience might be like, there was a clear determination to avoid inflicting this on others.

*“*…*it is really conflicting because sometimes I find it quite comforting*…*there's an option there to stop the pain. But* … *I always come out going ‘there's no way I can do this without affecting someone else, [it's] still not an option’”*. (Male, 40s, bereaved by the suicide of his friend in young adulthood).

###### Suicide being a permanent solution to a temporary problem

All participants had questioned why someone they knew well had chosen suicide, considering explanations such as struggles with mental health, work, or relationships. Although able to recognize how the deceased may have felt entrapped by their problems, three participants found it hard to accept that this was not a ‘*temporary, transient*’ and resolvable situation. As part of the process of weighing up suicide as a potential escape option, this frustration created a resolve to explore other solutions if ever similarly trapped, serving as a reminder that their problems were temporary.

*“It's all very much of that moment in time, I don't think the problems would have been, in the long‐term, insolvable.”* (Male, 60s, bereaved by the suicide of his daughter).

*“*…*[it's] desperate that they thought that was the only way out in that second”* (Female, 50s, bereaved by the suicide of her son).

These accounts also conveyed a poignancy in their sense of frustration that someone they loved had felt so trapped close by their situation.

##### Sub‐theme 1.3: Conflicting views on suicide as a potential option

Six participants expressed views coded under both sub‐themes 1.1 and 1.2 (see Table 2). In occupying both positions they described an uncomfortable tension; feeling drawn towards an option for escape yet concerned about the potential effect on others. This sense of conflict illustrated that there was no clear binary distinction between these perspectives, providing an insight into the ambivalence of the suicidal mind. Accounts revealed that at times the allure of escape could feel tempting but was countered by an awareness of the potentially catastrophic impact on others and the need to explore other problem‐solving approaches. One respondent encapsulated this difficult tension between the attraction of escape and the repulsion of impacting others:

"…*when you feel really down, you're very aware how much worse you can make it for other people, even if you escape. And would you do that to people that you love. If you love them, then no.”* (Female, 60s, bereaved by the suicide of her ex‐partner).

#### Theme 2: Impact of the method of suicide on consideration of own potential suicide methods

Nine participants shared reflections on which suicide method they might consider, which were clearly linked to the method used by the deceased. However, their greater awareness of technical aspects of this specific method had informed the views in ways that deterred them from using the same method.

##### Sub‐theme 2.1: Aversion to the same method

All nine participants described an emotional aversion to using the same method. Reasons most commonly related to aversive appraisals of the pain, damage or distress involved in attempting something similar.

*“*…*I would never be able to kill myself with the way [she] did because of the level of distress it would cause me”* (Female, 20s, bereaved by the suicide of her sister).

*“I'm just not brave enough to do something like that*…*I'm not brave enough to do what he did.”* (Female, 50s, bereaved by the suicide of her son).

Instead, a number had weighed up other suicide methods, particularly those that were deemed less violent, painful or distressing.

One outlier described a sense of his brother's ‘ownership’ of that suicide method and could never envisage himself using this out of a sense of respect that it ‘belonged’ to his brother.

##### Sub‐theme 2.2: A desire to protect others from the trauma of discovery

Four individuals were deterred from the method used by the deceased due to concerns about the impact on others. They ruminated on what those finding the body must have faced and were determined, should they ever attempt suicide, to minimize this trauma.

*“*…*the most I've thought about is the pain that he did to others. That's become quite an obsession for me*…*how would I do it having the minimum impact on others. And I've thought quite elaborate plans to try and avoid what happened in that situation, should I do it.”* (Male, 40s, bereaved by the suicide of his friend in young adulthood).

*“*…*I was so traumatized by what had happened to [her] and the way she looked afterwards*…*so I thought the best way for me is to make it easy for others when they come across me. To make it so that, I just looked like I'd fallen asleep*…*it was about just making [it] as least traumatic to other people. That I wasn't inconveniencing them.”* (Female, 40s, bereaved by the suicide of her adolescent daughter).

This sense of concern for others did not detract from the idea of attempting suicide per se, but indicated a clear preference for not using this same method.

##### Sub‐theme 2.3: Concerns around being found alive and wanting to guarantee own death

Five participants expressed a fear of surviving a suicide attempt, describing the need to identify a method “*so that I won't be able to come back*.*”* They described *“the risk of living*” and “*worse of all, not succeeding*.” This related to a determination to die and a dread of surviving yet sustaining debilitating injury.

*“It would be the not dying that would be the scary bit.”* (Female, 20s, bereaved by the suicide of her father).

In some cases, this also acknowledged a dread that first responders might have to attempt a distressing resuscitation.

*“*…*the only kind of definitive thing it's given is I would want to make sure I was dead. I would hate for somebody to find me and have to feel like they had to try and revive me.”* (Female, 20s, bereaved by the suicide of her father).

#### Theme 3: Experience of suicidal ideation as a means of understanding the deceased's suicidal mind

Fourteen participants described post‐bereavement suicidal ideation and for most this was their first experience of suicidal thoughts. Although frightening, some of the participants felt that experiencing passive or active suicidal thoughts helped them understand what their friend or relative might have been going through. When “*the penny dropped”* it helped address their agonizing questioning over why they might have chosen suicide, finally providing “*the answer that had kept me awake for months”*.

*"I think it took me to a very, very scary place that I don't ever, ever want to be in again but in some ways, I felt like, because I kept asking ‘why?’, the universe showed me why*… *And that's when I kind of went ‘Woah, I get it, I get it now. I understand why’.”* (Female, 40s, bereaved by the suicide of her friend).

Many participants described the intense sadness they had felt about someone they were close to ‘choosing’ to leave them behind, but how experiencing suicidal ideation themselves had given them a realization that a suicidal frame of mind limits one's apparent options and awareness of the likely impact on others.

*“I used to think it might have just been a very difficult decision for him, because he would have thought of everyone else. But*…*I know that that wouldn't have been part of his mindset because I've been there myself, you just don't*…*think of anyone else whatsoever. It's all about you.”* (Male, 20s, bereaved by the suicide of his brother).

Three participants had attempted suicide since the bereavement. This had provided insights into the intensity of acute suicidal distress, the realization that “*you just don't see how things can move on”* and “*you don't think of the stuff that you're leaving behind”* as well as the difficulties in seeking help at such points.

*“*…*when I tried to take my own life, it became blatantly obvious to me then, how [she] must have felt*…*I couldn't see another way out*…*and*…*I thought, ‘well I didn't turn to anyone, either, didn't want to burden them’. So, in some ways it was a useful experience*…*because it gave me a really deep understanding of why people take their own life.”* (Female, 40s, bereaved by the suicide of her adolescent daughter).

Such experiences of suicidal thinking appeared to have been distressing but were in some way valued for helping the bereaved comprehend the experiences of the deceased and the nature of their decision‐making processes. This had helped them “*understand that actually, she couldn't help it”* and to “*come to terms with that bit of grief, which is anger”*.

#### Theme 4: Thoughts related to reunion with the deceased

Six participants expressed a desire for reunion with the deceased, describing “*attractive*” urges to “*be with them*” that motivated suicidal intent.

*“When I was fully suicidal, a lot of it was* … *wanting to be with him.”* (Male, 20s, bereaved by the suicide of his brother).

*“It was a pull, that was one of the things on the balance that would say ‘This is a great idea’*…*‘Suicide's a good option because it means that I'll see [her] again’*…*I was going to stop the pain*…*seeing [her] again.”* (Female, 40s, bereaved by the suicide of her adolescent daughter).

For some this was a challenge to spirituality where individuals were unsure about their beliefs about the afterlife.

*“I don't know what I think about life after death*…*but I can still go in my head to a place of ‘well I'll be with them, that'll be better”* (Female, 20s, bereaved by the suicide of her sister).

The interplay of seeking escape and also yearning for reunion were viewed by some participants as particularly powerful influences motivating suicidal intent.

*“*…*it's a downward spiral because you want to be dead and you want to be with the other person and they're like ‘Do it, do it, do it’”* (Male, 20s, bereaved by suicide of his brother).

*“*…*the reuniting is a lot more connected to maybe the wanting to not be here”* (Female, 20s, bereaved by the suicide of her sister).

## DISCUSSION

### Main findings

Our analysis of interview data provides insights from a specific sample of 20 UK‐based suicide‐bereaved adults into how direct experience of suicide loss can impact upon a person's thoughts about their own suicide, including consideration of specific methods. All participants in this study described the devastating effects of the suicide on themselves and those around them. While some reported being deterred from suicide, for others it had become an acceptable option. A third group described a more conflicted picture, feeling drawn towards suicide yet fearing inflicting the same devastation on others. Whilst direct exposure to suicide had diminished the fear of death for some, in line with the theoretical construct of cognitive availability (Florentine & Crane, 2010), this did not apply to all, suggesting that *increased* cognitive availability of suicide cannot be assumed after suicide loss. Notably, all of those who discussed method choice described feeling deterred from considering the same method as the deceased, arising from a fear of pain or from a desire to reduce the impact on others. This conflicts with the idea that greater technical knowledge increases ‘psychological closeness’ to a given method.

### Results in the context of other studies

Our findings regarding suicide loss instilling a sense of suicide becoming a more tangible option are consistent with other qualitative work (Bell et al., 2015; Grimmond et al., 2019; Miklin et al., 2019; Pitman et al., 2017). Quantitative studies of adolescents and young adults have also described an association between exposure to suicidal behavior and more accepting views towards suicide (Abbott & Zakriski, 2014; Stein et al., 1992). In our study, a number of participants described being preoccupied by thoughts of reunion with the deceased as well as longing for escape: a particularly potent combination regarding suicidal intent. Yearning for the deceased is a common grief reaction (Bowlby & Parkes, 1970) particularly after suicide loss (Young et al., 2012), and it was clear that individuals in this sample actively considered suicide as a means to achieving this. However, it is also possible that suicidality after suicide loss arises due to the influences of heritability, chronic stress, disrupted attachments and psychiatric disorder (Hua et al., 2020).

Our findings regarding those who were deterred from choosing suicide are consistent with those of studies finding no elevated risk of suicide attempt in adolescents exposed to a peer's suicide, with explanations couched in the deterrent effect of observing the aftermath of suicide (Brent et al., 1992, 1993, 1996). Qualitative data supports this deterrent effect. Young men in Northern Ireland described exposure to the pain of their friends' suicidal crises as reinforcing a belief that suicide was unacceptable (Jordan et al., 2012). Australian men described the most common reason for being deterred from a suicide attempt was the consequences for their family (Shand et al., 2015).

Our findings regarding aversion to using the same method as the deceased conflicts with existing theories of cognitive availability; that gaining technical knowledge of a method might increase its perceived utility (Barber & Miller, 2014; Florentine & Crane, 2010; Sarchiapone et al., 2011). Other British empirical work exploring suicide method choice has described deterrents against specific methods as including fears of injury and survival and concerns about the impact on other people (Marzano et al., 2021).

### Strengths and limitations

To our knowledge, this is the first qualitative interview study to probe how suicide bereavement might influence the way an individual processes the idea of suicide, including method choice. Our sensitive approach, using a careful risk protocol, provided containment to interviewees, permitting detailed insights into the processing of direct experiences of suicide loss. Our use of online rather than in person interviews widened our geographical reach within the UK, but only to the digitally connected. The online setting may also have facilitated disclosure through reducing social desirability bias (Lobe et al., 2022). Although efforts were made to recruit a diverse sample, the sample was aged 22–64 years and predominantly female (80%) and White (90%), limiting insights on the young or the elderly and our ability to identify any patterning of themes by gender or ethnicity. Our sampling methods may have recruited those already in receipt of bereavement support, neglecting the experience of those who tend not to seek help. Our targeted advert identified those who had been preoccupied by suicide following the suicide bereavement, so our findings may not be representative of the experiences of all suicide‐bereaved people. These factors limit the transferability of our findings, and there is a need for similar qualitative work with under‐represented samples, particularly among men and ethnic minority groups.

### Clinical, theory, and research implications

Our findings provide clinical insights into the nature of grief and the emergence of suicidality after suicide loss, including an aversion to the same method. This is an important consideration when exploring risk in suicide‐bereaved individuals who are suicidal, requiring sensitive inquiry. Within this UK‐based sample we identified three distinct groups: those who had resolved to avoid suicide, those who were psychologically closer to the idea of suicide, and those conflicted between the two. This highlights the need for clinicians to inquire carefully about a suicide‐bereaved person's perspectives on suicide, including method choice, helping explore distress as well as risk.

These new findings contribute to suicide theory in demonstrating how exposure to suicide might lead to changes (reductions or increases) in the cognitive availability of suicide after a close contact's suicide and how this might augment or diminish acquired capability for suicide. Specific factors described (awareness of the devastating emotional impact of suicide loss; resolution to seek other solutions; desire for reunion with the deceased; reduced fear of death) could be seen to influence capability for suicide in different ways. Further qualitative work in other samples would check consistency and provide other insights. The suggested pathways would then require empirical testing, using validated measures of these constructs, to inform theoretical models of cognitive availability.

Empirical research is needed to track the cognitive availability of suicide in a representative sample of suicide‐bereaved individuals from the point of direct exposure, including attitudes to specific methods. This should account for indirect influences from the media and cultural norms, and variation by age, gender, closeness to the deceased, or experiences of discovery. Such work is reliant on a better understanding of the boundary conditions for the construct of cognitive availability of suicide and the development of a validated measure of this construct. Interventional research is also needed to understand how to modify the cognitive availability of suicide in those directly exposed to suicide loss. Such psychological interventions are likely to require a high degree of skill in exploring complex and distressing cognitions whilst avoiding mental rehearsal of suicidal behavior that might further increase cognitive availability. Such interventions have the potential to reduce suicide risk whilst also reducing the distress associated with suicidality; an advantage over physical restriction of means.

## CONCLUSION

This qualitative study of 20 suicide‐bereaved adults describes how direct exposure to the trauma of suicide loss influences a bereaved person's thoughts about suicide in divergent ways. Some felt drawn towards suicide as an option, whilst some were deterred from it, and others remained conflicted. This work develops our understanding of the theoretical constructs of cognitive availability of suicide and acquired capability for suicide, identifying areas for further investigation.

## FUNDING INFORMATION

The authors received no specific funding for this work.

## CONFLICT OF INTEREST STATEMENT

AP is a patron of the Support After Suicide Partnership. All other authors state that they have no other conflicts of interest.

## Supporting information


Appendix S1.

Appendix S2.

Appendix S3.

## Data Availability

To protect confidentiality the UCL Research Ethics Committee has restricted access to the pseudonymised interview transcripts stored on the university's Data Safe Haven so that they are only accessible to members of the research team. Other researchers can apply formally to join the team to analyze data to address a specific research question. The rationale for this is that the data contain potentially identifying or sensitive information about bereaved individuals and public access risks identifying them more widely.

## References

[sltb13134-bib-0001] Abbott, C. H. , & Zakriski, A. L. (2014). Grief and attitudes toward suicide in peers affected by a cluster of suicides as adolescents. Suicide & Life‐Threatening Behavior, 44(6), 668–681. 24806293 10.1111/sltb.12100

[sltb13134-bib-0002] Barber, C. W. , & Miller, M. J. (2014). Reducing a suicidal person's access to lethal means of suicide: A research agenda. American Journal of Preventive Medicine, 47(3), S264–S272. 25145749 10.1016/j.amepre.2014.05.028

[sltb13134-bib-0003] Bell, J. , Stanley, N. , Mallon, S. , & Manthorpe, P. J. (2015). Insights into the processes of suicide contagion: Narratives from young people bereaved by suicide. Suicidology Online, 6(1), 43–52.

[sltb13134-bib-0004] Biddle, L. , Gunnell, D. , Owen‐Smith, A. , Potokar, J. , Longson, D. , Hawton, K. , Kapur, N. , & Donovan, J. (2012). Information sources used by the suicidal to inform choice of method. Journal of Affective Disorders, 136(3), 702–709. 22093678 10.1016/j.jad.2011.10.004

[sltb13134-bib-0005] Bowlby, J. , & Parkes, C. (1970). Separation and loss within the family. In E. J. Anthony (Ed.), The child in his family. Whiley.

[sltb13134-bib-0006] Braun, V. , & Clarke, V. (2006). Using thematic analysis in psychology. Qualitative Research in Psychology, 3(2), 77–101.

[sltb13134-bib-0007] Braun, V. , & Clarke, V. (2019). Reflecting on reflexive thematic analysis. Qualitative Research in Sport, Exercise and Health, 11(4), 589–597.

[sltb13134-bib-0008] Braun, V. , & Clarke, V. (2021a). One size fits all? What counts as quality practice in (reflexive) thematic analysis? Qualitative Research in Psychology, 18(3), 328–352.

[sltb13134-bib-0009] Braun, V. , & Clarke, V. (2021b). To saturate or not to saturate? Questioning data saturation as a useful concept for thematic analysis and sample‐size rationales. Qualitative Research in Sport, Exercise and Health, 13(2), 201–216.

[sltb13134-bib-0010] Brent, D. A. , Moritz, G. , Bridge, J. , Perper, J. , & Canobbio, R. (1996). Long‐term impact of exposure to suicide: A three‐year controlled follow‐up. Journal of the American Academy of Child and Adolescent Psychiatry, 35(5), 646–653. 8935212 10.1097/00004583-199605000-00020

[sltb13134-bib-0011] Brent, D. A. , Perper, J. , Moritz, G. , Allman, C. , Friend, A. M. , Schweers, J. O. , Roth, C. , Balach, L. , & Harrington, K. (1992). Psychiatric effects of exposure to suicide among the friends and acquaintances of adolescent suicide victims. Journal of the American Academy of Child and Adolescent Psychiatry, 31(4), 629–639. 1644725 10.1097/00004583-199207000-00009

[sltb13134-bib-0012] Brent, D. A. , Perper, J. A. , Moritz, G. , Allman, C. , Schweers, J. O. , Roth, C. , Balach, L. , Canobbio, R. , & Liotus, L. (1993). Psychiatric sequelae to the loss of an adolescent peer to suicide. Journal of the American Academy of Child and Adolescent Psychiatry, 32(3), 509–517. 8496113 10.1097/00004583-199305000-00004

[sltb13134-bib-0013] Bridge, J. A. , Greenhouse, J. B. , Ruch, D. , Stevens, J. , Ackerman, J. , Sheftall, A. H. , Horowitz, L. M. , Kelleher, K. J. , & Campo, J. V. (2020). Association between the release of Netflix's 13 reasons why and suicide rates in the United States: An interrupted time series analysis. Journal of the American Academy of Child and Adolescent Psychiatry, 59(2), 236–243. 31042568 10.1016/j.jaac.2019.04.020 PMC6817407

[sltb13134-bib-0014] Cerel, J. , Brown, M. M. , Maple, M. , Singleton, M. , Van de Venne, J. , Moore, M. , & Flaherty, C. (2019). How many people are exposed to suicide? Not Six. Suicide and Life‐Threatening Behavior, 49(2), 529–534. 29512876 10.1111/sltb.12450

[sltb13134-bib-0015] Clark, S. E. , & Goldney, R. (2000). The impact of suicide on relatives and friends. In K. Hawton & K. Van Heeringen (Eds.), The international handbook of suicide and attempted suicide (pp. 467–484). Wiley.

[sltb13134-bib-0016] Creswell, J. W. , & Miller, D. L. (2000). Determining validity in qualitative inquiry. Theory into Practice, 39(3), 124–130.

[sltb13134-bib-0017] Dempsey, R. C. , Fedorowicz, S. E. , & Wood, A. M. (2023). The role of perceived social norms in non‐suicidal self‐injury and suicidality: A systematic scoping review. PLoS One, 18(6), e0286118. 37352219 10.1371/journal.pone.0286118 PMC10289472

[sltb13134-bib-0020] Erlangsen, A. , Runeson, B. , Bolton, J. M. , Wilcox, H. C. , Forman, J. L. , Krogh, J. , Shear, M. K. , Nordentoft, M. , & Conwell, Y. (2017). Association between spousal suicide and mental, physical, and social health outcomes: A longitudinal and nationwide register‐based study. JAMA Psychiatry, 74(5), 456–464. 28329305 10.1001/jamapsychiatry.2017.0226 PMC5470398

[sltb13134-bib-0021] Florentine, J. B. , & Crane, C. (2010). Suicide prevention by limiting access to methods: A review of theory and practice. Social Science & Medicine, 70(10), 1626–1632. 20207465 10.1016/j.socscimed.2010.01.029

[sltb13134-bib-0023] Galbin, A. (2014). An introduction to social constructivism. Social Research Reports, 6(26), 82–92.

[sltb13134-bib-0024] Grimmond, J. , Kornhaber, R. , Visentin, D. , & Cleary, M. (2019). A qualitative systematic review of experiences and perceptions of youth suicide. PLoS One, 14(6), e0217568. 31188855 10.1371/journal.pone.0217568 PMC6561633

[sltb13134-bib-0026] Hill, N. T. , Robinson, J. , Pirkis, J. , Andriessen, K. , Krysinska, K. , Payne, A. , Boland, A. , Clarke, A. , Milner, A. , Witt, K. , & Krohn, S. (2020). Association of suicidal behavior with exposure to suicide and suicide attempt: A systematic review and multilevel meta‐analysis. PLoS Medicine, 17(3), e1003074. 32231381 10.1371/journal.pmed.1003074 PMC7108695

[sltb13134-bib-0027] Hua, P. , Maple, M. , Hay, K. , & Bugeja, L. (2020). Theoretical frameworks informing the relationship between parental death and suicidal behaviour: A scoping review. Heliyon, 6(5), e03911. 32426539 10.1016/j.heliyon.2020.e03911 PMC7226651

[sltb13134-bib-0028] Joiner, T. E. (2005). Why people die by suicide. Harvard University Press.

[sltb13134-bib-0029] Jordan, J. , McKenna, H. , Keeney, S. , Cutcliffe, J. , Stevenson, C. , Slater, P. , & McGowan, I. (2012). Providing meaningful care: Learning from the experiences of suicidal young men. Qualitative Health Research, 22(9), 1207–1219. 22785623 10.1177/1049732312450367

[sltb13134-bib-0032] Lobe, B. , Morgan, D. , & Hoffman, K. (2022). A systematic comparison of in‐person and video‐based online interviewing. International Journal of Qualitative Methods, 21, 1–12.

[sltb13134-bib-0033] Marzano, L. , Katsampa, D. , Mackenzie, J. M. , Kruger, I. , El‐Gharbawi, N. , Ffolkes‐St‐Helene, D. , Mohiddin, H. , & Fields, B. (2021). Patterns and motivations for method choices in suicidal thoughts and behaviour: Qualitative content analysis of a large online survey. BJPsych Open, 7(2), e60. 33622447 10.1192/bjo.2021.15 PMC8058851

[sltb13134-bib-0035] Miklin, S. , Mueller, A. S. , Abrutyn, S. , & Ordonez, K. (2019). What does it mean to be exposed to suicide?: Suicide exposure, suicide risk, and the importance of meaning‐making. Social Science & Medicine, 1(233), 21–27. 10.1016/j.socscimed.2019.05.019 31153084

[sltb13134-bib-0036] Niederkrotenthaler, T. , Braun, M. , Pirkis, J. , Till, B. , Stack, S. , Sinyor, M. , Tran, U. S. , Voracek, M. , Cheng, Q. , Arendt, F. , Scherr, S. , Yip, P. S. F. , & Spittal, M. J. (2020). Association between suicide reporting in the media and suicide: Systematic review and meta‐analysis. BMJ, 18, 368. 10.1136/bmj.m575 PMC7190013 32188637

[sltb13134-bib-0037] O'Connor, R. C. , & Kirtley, O. J. (2018). The integrated motivational–volitional model of suicidal behaviour. Philosophical Transactions of the Royal Society, Biological Sciences, 373(1754), 20170268. 30012735 10.1098/rstb.2017.0268 PMC6053985

[sltb13134-bib-0039] Pirkis, J. , & Blood, R. W. (2001). Suicide and the media: Part II. Portrayal in fictional media. Crisis: The Journal of Crisis Intervention and Suicide Prevention, 22(4), 155–162. 10.1027//0227-5910.22.4.155 11848659

[sltb13134-bib-0040] Pitman, A. , Nesse, H. , Morant, N. , Azorina, V. , Stevenson, F. , King, M. , & Osborn, D. (2017). Attitudes to suicide following the suicide of a friend or relative: A qualitative study of the views of 429 young bereaved adults in the UK. BMC Psychiatry, 17(1), 1–11. 29237447 10.1186/s12888-017-1560-3 PMC5729247

[sltb13134-bib-0041] Pitman, A. , Osborn, D. , King, M. , & Erlangsen, A. (2014). Effects of suicide bereavement on mental health and suicide risk. The Lancet Psychiatry, 1(1), 86–94. 26360405 10.1016/S2215-0366(14)70224-X

[sltb13134-bib-0042] Pitman, A. L. , Osborn, D. P. , Rantell, K. , & King, M. B. (2016). Bereavement by suicide as a risk factor for suicide attempt: A cross‐sectional national UK‐wide study of 3432 young bereaved adults. BMJ Open, 6(1), e009948. 10.1136/bmjopen-2015-009948 PMC4735143 26813968

[sltb13134-bib-0043] Posselt, M. , McIntyre, H. , & Procter, N. (2021). The impact of screen media portrayals of suicide on viewers: A rapid review of the evidence. Health & Social Care in the Community, 29(1), 28–41. 32716609 10.1111/hsc.13112

[sltb13134-bib-0019] Public Health England National Suicide Prevention Alliance . (2015). Help is at Hand: Support after someone may have died by suicide. [Internet]. Support After Suicide Partnership: https://supportaftersuicide.org.uk/resource/help‐is‐at‐hand/

[sltb13134-bib-0044] QSR International . (2018). NVivo qualitative data analysis software [computer program]. In Version 12. QSR International. Available from: https://www.qsrinternational.com/nvivo‐qualitative‐data‐analysis‐software/home

[sltb13134-bib-0045] Quayle, K. , Jones, P. , Di Simplicio, M. , Kamboj, S. , & Pitman, A. (2023). Exploring the phenomenon of intrusive mental imagery after suicide bereavement: A qualitative interview study in a British sample. PLoS One, 18(8), e0284897. 37590210 10.1371/journal.pone.0284897 PMC10434947

[sltb13134-bib-0046] Rogers, M. L. , Hom, M. A. , Stanley, I. H. , & Joiner, T. E. (2019). Brief measures of physical and psychological distance to suicide methods as correlates and predictors of suicide risk: A multi‐study prospective investigation. Behaviour Research and Therapy, 1(120), 103330. 10.1016/j.brat.2018.11.001 30448268

[sltb13134-bib-0047] Sarchiapone, M. , Mandelli, L. , Iosue, M. , Andrisano, C. , & Roy, A. (2011). Controlling access to suicide means. International Journal of Environmental Research and Public Health, 8(12), 4550–4562. 22408588 10.3390/ijerph8124550 PMC3290984

[sltb13134-bib-0048] Schlichthorst, M. , Reifels, L. , Spittal, M. , Clapperton, A. , Scurrah, K. , Kolves, K. , Platt, S. , Pirkis, J. , & Kyrsinska, K. (2022). Evaluating the effectiveness of components of National Suicide Prevention Strategies: An interrupted time series analysis. Crisis, 44(4): 318‐328. 36537610 10.1027/0227-5910/a000887

[sltb13134-bib-0050] Shand, F. L. , Proudfoot, J. , Player, M. J. , Fogarty, A. , Whittle, E. , Wilhelm, K. , Hadzi‐Pavlovic, D. , McTigue, I. , Spurrier, M. , & Christensen, H. (2015). What might interrupt men's suicide? Results from an online survey of men. BMJ Open, 5(10), e008172. 10.1136/bmjopen-2015-008172 PMC4611172 26474936

[sltb13134-bib-0051] Stein, D. , Witztum, E. , Brom, D. , De‐Nour, A. K. , & Elizur, A. (1992). The association between adolescents' attitudes toward suicide and their psychosocial background and suicidal tendencies. Adolescence, 1, 959. 1471571

[sltb13134-bib-0053] Tidemalm, D. , Runeson, B. , Waern, M. , Frisell, T. , Carlström, E. , Lichtenstein, P. , & Långström, N. (2011). Familial clustering of suicide risk: A total population study of 11.4 million individuals. Psychological Medicine, 41(12), 2527–2534. 21733212 10.1017/S0033291711000833 PMC3207221

[sltb13134-bib-0054] Tong, A. , Sainsbury, P. , & Craig, J. (2007). Consolidated criteria for reporting qualitative research (COREQ): A 32‐item checklist for interviews and focus groups. International Journal for Quality in Health Care, 19(6), 349–357. 17872937 10.1093/intqhc/mzm042

[sltb13134-bib-0056] Van Orden, K. A. , Witte, T. K. , Cukrowicz, K. C. , Braithwaite, S. R. , Selby, E. A. , & Joiner, T. E., Jr. (2010). The interpersonal theory of suicide. Psychological Review, 117(2), 575–600. 20438238 10.1037/a0018697 PMC3130348

[sltb13134-bib-0058] World Health Organisation . (2021). Suicide rates. Available from: https://www.who.int/news‐room/fact‐sheets/detail/suicide

[sltb13134-bib-0060] Young, I. T. , Iglewicz, A. , Glorioso, D. , Lanouette, N. , Seay, K. , Ilapakurti, M. , & Zisook, S. (2012). Suicide bereavement and complicated grief. Dialogues in Clinical Neuroscience, 14(2), 177–186. 22754290 10.31887/DCNS.2012.14.2/iyoung PMC3384446

