# Supplementary material for: Understanding the influence of suicide bereavement on the cognitive availability of suicide: Qualitative interview study of UK adults
Source: Suicide Life Threat Behav. 2024 Nov 4;55(1):e13134. doi: 10.1111/sltb.13134 (PMC11716337; doi:10.1111/sltb.13134)
Supplement: Supplementary file 1 — Appendix S1. [file SLTB-55-0-s002.docx]

**Appendix 1: Topic guide**

At the start of each interview the interviewer will be clear that the interviewee does not have to answer any of the questions if they prefer not to, and that in this event the interviewer will move onto the next question, and will fully understand their preference not to respond. The interviewer will also be clear that they can pause the interview at any point. Before every few questions the interviewer will remind the interviewee that they don’t have to answer any question if they prefer not to.

The interviewer will begin with fairly broad questions, with some initial orienting questions to build on what was disclosed in the online screening questionnaire. The purpose of these will also be to build rapport, before moving on to a deeper exploration of imagery and/or cognitions.

*Opening statement, e.g. “You mentioned to us in the questionnaire that you lost your [insert kinship] due to suicide [X] years ago, and that he/she was [X] years old at the time” …*

- *How would you describe your relationship with [name]?*
- *How did you find out about the death? Were there aspects of it that you did not find out about until much later?*
- *Are you aware of any factors or circumstances that may have contributed to their death? (prompts: including any mental health difficulties, financial difficulties, significant life events).*
- *Did you have any warning signs that he/she was considering suicide? Eg expressing suicidal thoughts, previous attempts, comments on social media, becoming very withdrawn.*
- *Have you had thoughts about how they might have felt before they died? What are your thoughts about what was going on in their mind at the time/leading up to his/her death?*
- (If the nature of the nature of the death was established from information gathered on the screening questionnaire in the interview) *You mentioned your [kinship/name] died in a peaceful way/violent way, do you have any thoughts on what might have influenced the method that s/he might have chosen?*

or

- (if no prior information disclosed) *Would you mind if I asked about the way in which [name/kinship] died? And I ask you this because I would be interested to know why you thought they might have chosen that particular method.*
- *In what way, if any, has their death impacted/affected your thoughts about suicide? Has this affected your own thoughts about how you cope with difficulties in your own life?*
- *Have you had thoughts about wanting to be with that person? Do you have thoughts about reunion/reuniting with [name]?*

If they mentioned they had experienced their own suicidal thoughts:

- *You mentioned that you experienced your own suicidal thoughts following the death of [name], how has the way in which [name] died impacted upon you? (Prompt for thoughts, feelings and behaviours)*
- *How frequently do you experience these thoughts? How compelling are they? (prompt/clarification): do you feel they influence your thoughts or actions in any way? How do you cope with this?*
