# Supplementary material for: Understanding the influence of suicide bereavement on the cognitive availability of suicide: Qualitative interview study of UK adults
Source: Suicide Life Threat Behav. 2024 Nov 4;55(1):e13134. doi: 10.1111/sltb.13134 (PMC11716337; doi:10.1111/sltb.13134)
Supplement: Supplementary file 2 — Appendix S2. [file SLTB-55-0-s003.docx]

**Appendix 2: Risk Protocol**

If participants are deemed to be at risk due to suicidal thoughts, contact with the GP will be made following discussion with and ideally with full consent from the participant. If the participant refuses permission for the researcher to inform the GP, then the researcher will immediately consult the Supervisory Clinician who will consider the participant’s situation and, if necessary, assess the participant. If it is concluded that there is a significant risk, the participant’s GP will be notified with or without the participant’s consent. If the risk is severe and urgent, the Supervisory Clinician/researcher will contact the emergency services without first assessing the participant. In these cases, the decision will be explained to the participant as soon as possible.

It is explained to participants in the study’s information sheet that the researcher may need to pass on information about potential risk of harm, and the consent form requests their GP contact information, so enacting this protocol would not be a surprise to participants. Researchers may initiate the risk protocol each time a participant expresses current suicidal thoughts or thoughts of harm to self or others at any point of contact with the researcher.

Use of the risk protocol will be undertaken with appropriate supervision. The Primary Study Supervisor has overall responsibility for risk assessment and management for this study. The Primary Study Supervisor is responsible for:

1. ensuring that supervision for risk issues is accessible and readily available.
2. ensuring that any researchers involved in this study are adequately trained in the use of the risk protocol prior to participant contact.
3. ensuring that researchers are aware of who can provide supervision (i.e. the Supervisory Clinicians) and how supervisors can be contacted.

**Action**

Before conducting the interviews, researchers should ensure that the contact details for Study Supervisors, Supervisory Clinicians and the participant’s GP details are current. Although encouraged, it is not mandatory for participants to provide GP details, as some individuals may not be registered with a GP. If risk issues were to arise in relation to a participant who has not provided GP details, these have been considered below.

Throughout the interview, the researcher will be aware of any indications of potential risk including suggestions that they may harm themselves or others, or indications that they are struggling to cope with their life, such as phrases like *“I’m not sure if I can cope any more”* or *“I wish it would all end”.*

If the researcher notices any of these indicators, they will pause the interview at an appropriate point and return to any comments made by the participant to explore them further. The researcher will ask appropriate questions to assess how immediate the risk is to the participant or others. This will involve assessing whether the participant has made any plans for action, and how immediate these plans are.

Example phrasing:

*“I’m struck by how difficult you’ve found the loss of X. Bereavement, particularly bereavement by suicide can be really challenging to cope with, so it’s understandable that you’ve been having some difficult thoughts. Earlier, you mentioned that *summarise what participant said*, could you tell me more about that?”*

*“You’ve spoken about your thoughts to harm yourself/somebody else; can I ask if you have made any plans to do so?”*

*“When have you thought you might do this?”*

Depending on the participant’s responses to the researcher’s questions, one of three actions will be taken:

**1. If the participant has thoughts about harming themselves/somebody else but no plans:**

Non-urgent action will be taken.

The researcher will suggest that the participant contacts their GP and remind them of the list of support organisations that has been provided to them.

Example wording:
“*From what you’ve said, it seems like you’re finding it difficult to cope with your bereavement with the support you currently have. I’d recommend speaking to your GP about what’s happened and how you’re feeling so that they can arrange professional support for you, if it’s appropriate and if that’s what you want. At the end of the session, I’ll give you a list of resources and charities who help those who have been bereaved; this gives you information about how you can access support groups, phone lines and online resources that you might also find useful.”*

**2. If the participant has significant plans to harm themselves/somebody else that are not immediate:**

Non-urgent action will be taken.

The researcher will explain to the participant they will pause the interview to confer with the supervisory clinician. They will then call the supervisory clinician to inform them of the situation and to confirm further action is appropriate. If the clinician believes contacting a medical professional is appropriate, the researcher will aim to gain the participant’s permission before doing so, but will make contact even without.

If GP contact details are provided by the participant on the consent form, the researcher will call them. On telephoning, if the participant’s GP is not available then the researcher should ask to speak to the duty doctor. If the participants’ thoughts are about harming somebody else, the researcher will also call 101 (the non-emergency police phone line) to ensure they aware of the risk.

If no medical professionals at the practice are currently available or if the participant has not provided GP details, the researcher will call 111 (the non-emergency healthcare service line). The researcher should make it clear that no clinical risk assessment has been performed and that clinical responsibility for the study participants remains with the medical professionals.

If the participants’ thoughts are about harming somebody else, the researcher will also call 101 (the non-emergency police phone line) to ensure they aware of the risk.

Example wording:

*“From what you’ve said, it seems like you’re finding it difficult to cope with your bereavement with the support you currently have. I’m going to make some phone calls to your GP to let them know how you are feeling so that they can arrange some help for you. I understand that this could be scary for you, but I will let you know what’s happening as soon as I’ve spoken to them.”*

The risk protocol form will be completed and stored securely to record that a risk protocol has been enacted.

**3. If the participant has plans to harm themselves/somebody else that are immediate:**

Urgent action will be taken.

The researcher will explain to the participant they will pause the interview to confer with the supervisory clinician. They will then call the supervisory clinician to inform them of the situation and to confirm further action is appropriate. If the clinician believes contacting a medical professional is appropriate, the researcher will aim to gain the participant’s permission before doing so, but will make contact even without.

The researcher will call 999 to relay the information provided by the participant and act according to guidance from the emergency services. The researcher should make it clear that no clinical risk assessment has been performed and that clinical responsibility for the study participants remains with the medical professionals.

If the researcher is concerned about their personal safety, they will leave the location.

Example wording:

*“From what you’ve said, it seems like you’re finding it difficult to cope with your bereavement with the support you currently have. I’m going to make some phone calls to the emergency services to let them know how you are feeling so that they can arrange some immediate help for you. I understand that this could be scary for you, but I will let you know what’s happening as soon as I’ve spoken to them.”*

The risk protocol form will be completed and stored securely to record that a risk protocol has been enacted.
