# Supplementary material for: Understanding the influence of suicide bereavement on the cognitive availability of suicide: Qualitative interview study of UK adults
Source: Suicide Life Threat Behav. 2024 Nov 4;55(1):e13134. doi: 10.1111/sltb.13134 (PMC11716337; doi:10.1111/sltb.13134)
Supplement: Supplementary file 3 — Appendix S3. [file SLTB-55-0-s001.docx]

**Appendix 3 – Supplementary quotes**

This supplementary file provides the full dataset of quotes coded under each theme.

Black text denotes quotes featuring in the main manuscript.

Blue text denotes quotes not provided in main manuscript and those sections of quotes not used where a shorter version of the quote is provided in the main manuscript.

Redactions of quotes are marked and indicate where excessive detail was provided of the method, as per media guidelines on the reporting of suicide.

1. **Divergent changes in views about suicidal as an option**
   1. **Suicide becomes an option**
      1. *Suicide being a legitimate option*

*“If it’s so unbearable, if this happens or that happens, I can always do that. It’s almost your get-out card, in a way. Afterwards, it’s like, which you would never have considered before, but it’s like, now, well there’s always that.”* (Female, 60s, bereaved by the suicide of her ex-partner)

*“…for me the suicidality was something after [she] died, I think it just, it becomes so much more of an option.”* (Female, 20s, bereaved by the suicide of her sister)

*“I think the other thing it did was, it made it an option. It was almost like, well, you know what, if she’s done it, then what’s to stop me doing it?...that seemed like a viable option to me.”*  (Female, 40s, bereaved by the suicide of her adolescent daughter)

*“…you miss them and you want, like I’ve wanted to put his clothes on. I’ve worn his jumper, or, you want to be with him again and in wanting to be with him again, you can want to do anything that he might have done, which might include all of those things he did at the end.”* (Female, 40s, bereaved by the suicide of her brother)

*“I guess I partially feel more like, I partially feel like it makes it almost like a valid option”* (Female, 20s, bereaved by the suicide of her father)

*“But then as I became more ill in the time after his death, it seemed like a very very viable option for me. Very viable.”* (Male, 20s, bereaved by the suicide of his brother)

*“I slightly have this perspective of like, it would always be there as a back-up, the option to die is never going anywhere, you know, you’ve always got the option to live and the option to die.”* (Female, 20s, bereaved by the suicide of her father)

*“…it kind of subconsciously is like ‘oh right, yes, suicide is a thing, that, you know, could happen, is something I could do’…‘I don’t really care about life’, I’m like ‘oh well, suicide is always an option’.* (Female, 20s, bereaved by the suicide of her father)

*“It is an option. If things got really, really, really bad, I’ve always got that. So you jump to that”* *(*Female, 60s, bereaved by the suicide of her ex-partner)

*“But it’s always that thing of, if you lose the people that make your life worth something, then you know that would be probably the option. But unless that happened, it’s not an option.”* (Female, 60s, bereaved by the suicide of her ex-partner)

- - 1. *Normalised dying by suicide*

*“I think because [she] had done it, it almost normalised it for me.”* (Female, 40s, bereaved by the suicide of her adolescent daughter)

*“It’s completely normalised it. It’s f****** everywhere.”* (Male, 20s, bereaved by the suicide of his brother)

*“…it becomes a reality, it becomes something that people do.”* (Female, 20s, bereaved by the suicide of her sister)

*“I guess it’s that kind of, you know, ‘not somebody I know’ thing, you don’t really think about what happens when you cross the road, unless you know somebody that’s been hit by a car, I don’t know, I guess it feels like, up until that point, I’d never known anybody who’d killed themselves, or known anybody who’d known anybody that’d killed themselves, and it almost just felt like it was something that, you know, famous people died of drug overdoses, or, you know, it felt like something very not real to me, and I guess, now it does feel very real to me, the same way that the risk of dying of cancer feels more real to me, and I guess probably that’s what I mean by it, and I don’t know, I guess similarly, I feel like I’m, you know, I don’t know, this is why it’s a contradiction, I guess contrary to what I was just saying, whilst I would never be somebody who would [details of method redacted] like somebody on TV, you know, neither was my Dad and he tried to take his own life and did it, so it does make it feel slightly more like, well, it can happen and it can be done by normal people.”* (Female, 20s, bereaved by the suicide of her father)

*“I think the other thing it did was, it made it an option. It was almost like, well, you know what, if she’s done it, then what’s to stop me doing it?”* (Female, 40s, bereaved by the suicide of her adolescent daughter)

- - 1. *The comfort of having suicide as an option*

*“Because it is really conflicting because sometimes I find it quite comforting, that you know, there’s an option there to stop the pain, as it were.”* (Male, 40s, bereaved by the suicide of his friend in young adulthood)

*“…and there’s always that it feels like a comfort to know that there’s that fall back to where I’ve already decided like if things get too bad, I now know there’s an answer to do it”* (Female, 40s, bereaved by the suicide of her brother and her cousin)

*“That’s when I’m in a suicidal state, that I have a different method and a different set but it’s the same sense of ‘it’s there’, it’s a comfort.”* (Female, 20s, bereaved by the suicide of her sister)

*“I think it was a comforting thought. I didn’t ever feel like frightened of it or anything, like I was never frightened of it. I used to like, I used to worry sometimes about how much I was, what’s the word when you’re like, not fearless of it, like I always used to think, I dunno how I managed to stop myself from doing it, because the urge was so high.”* (Female, 20s, bereaved by the suicide of her boyfriend)[Files\\EB](file:///C:\Users\poppyjones\Documents\DClinPsy\Thesis\Part%202%20-%20Research%20Paper\Analysis\c54ef3b6-09b3-472b-88d1-48883464d40c)0 references coded, 0.00% coverage[Files\\JD](file:///C:\Users\poppyjones\Documents\DClinPsy\Thesis\Part%202%20-%20Research%20Paper\Analysis\3a8105dd-dbf3-4f2a-a968-8bae054f822e)

*“It’s almost like…if I got to 50 and things were awful, well I could always take my own life then”* (Female, 20s, bereaved by the suicide of her father)

*“… when you’re feeling really low. When you’re sort of struggling with something. When the stress is high…That has shifted, so I can feel really low and you can still think of it as an option, but you know it’s not an option that you need now. Do you see what I mean? It’s more like, oh if the worst happens, then there’s always….”* (Female, 60s, bereaved by the suicide of her ex-partner)

“…*it’s made my thought of suicide, well you know what, if I was in a terrible situation, maybe I’d just take my own life*.” (Female, 40s, bereaved by the suicide of her adolescent daughter)

- - 1. *Reduced fear of death*

*“…one of the things that has persisted is it’s made me a little bit less scared of death. And whilst I’m fine now, I do look at it and wonder if that would impact again if I felt down.”* (Female, 40s, bereaved by the suicide of her adolescent daughter)

*“As in the fear of death is not that big anymore.”* (Male, 50s, bereaved by the suicide of his adolescent son)

*“I’m not scared of dying.”* (Female, 50s, bereaved by the suicide of her son)

*“I wasn’t scared about dying.”* (Male, 20s, bereaved by the suicide of his brother)

*“…and then obviously [deceased’s name] dying, kind of ended up with my wanting to die myself and not being scared of death.”* (Male, 20s, bereaved by the suicide of his brother)

*“I think it was a comforting thought. I didn’t ever feel like frightened of it or anything, like I was never frightened of it. I used to like, I used to worry sometimes about how much I was, what’s the word when you’re like, not fearless of it, like I always used to think, I dunno how I managed to stop myself from doing it, because the urge was so high.”* (Female, 20s, bereaved by the suicide of her boyfriend)[Files\\EB](file:///C:\Users\poppyjones\Documents\DClinPsy\Thesis\Part%202%20-%20Research%20Paper\Analysis\c54ef3b6-09b3-472b-88d1-48883464d40c)0 references coded, 0.00% coverage[Files\\JD](file:///C:\Users\poppyjones\Documents\DClinPsy\Thesis\Part%202%20-%20Research%20Paper\Analysis\3a8105dd-dbf3-4f2a-a968-8bae054f822e)

- 1. **Deterred from suicide being an option**
     1. *Not wanting to inflict the same suffering on others*

*“I don’t think, from having seen the mess, I wouldn’t want to put my wife through the mess that I’ve had to pick up with my children. I think it leaves [a] horrible legacy for the people left behind, that’s difficult to come to terms with, having witnessed this, I wouldn’t want to put them through it.”* (Male, 60s, bereaved by the suicide of his daughter)

*“I think part of why it never moved into what I would call active thoughts of it, was because I understood what it meant to do that to other people. So I think that probably was a block in my head to be able to actually ever have any actual thoughts of doing it because I very much understood from seeing like, well from knowing how I felt and also seeing all of my family, so I think from that point of view, that probably played in a lot as to how I actually felt about it.”* (Female, 20s, bereaved by the suicide of her mother)

*“Because it is really conflicting, because sometimes I find it quite comforting, that you know, there’s an option there to stop the pain, as it were. And then, I mean it is difficult, but I think that kind of processing the options, looking at the different paths, I think in some ways, it’s helpful, because what it does, because eventually I always come out going ‘there’s no way I can do this without affecting someone else, [it’s] still not an option’ and I think in a way, it’s quite helpful.”* (Male, 40s, bereaved by the suicide of his friend in young adulthood)

*“…it did make me realise that actually suicide isn’t a problem solved, it’s a problem created for others. Might have been solved for my daughter, but for others it is ongoing.”* (Male, 60s, bereaved by the suicide of his daughter)

*“Yeah, so once she had [died], I then, for the first time I guess, even had a background thought of ‘well that would be a way to escape all of this pain that she’s caused me because she’d done it’, but because of the very fact that she’s done it, also means that I can’t do it because I’ve seen what it’s done to everyone else and because I feel like I’ve not got these additional responsibilities that she had.”* (Female, 20s, bereaved by the suicide of her mother)

*“…it definitely was made me think a lot harder about what the impact would, you would leave on somebody and how awful that would be, I guess, having had to go through that myself.”* (Female, 20s, bereaved by the suicide of her father)

*“Even though I thought a lot about the pain going away, and that was the motivator for being like, I’ve literally, I’ve had enough. I always had to, like, I feel like I always had to stop myself because I always thought about, you know, ‘how hard has this been for you, can you imagine how hard this would be for my sister’”* (Female, 20s, bereaved by the suicide of her boyfriend)

*“…because you’ve been there…there is, I suppose, weirdly enough…there’s an upside. In that you’re very aware when you feel really down, you’re very aware how much worse you can make it for other people, even if you escape. And would you do that to people that you love. If you love them, then no.”* (Female, 60s, bereaved by the suicide of her ex-partner)

*“…I could never do it myself…I’ve got kids, I couldn’t do it to them, and I’ve got grandsons and I just, there’s thinking about it and there’s actually doing it”* (Female, 50s bereaved by the suicide of her husband)

*“I think it’s made me think about suicide quite a lot but it’s been overall a protective thing because I think I keep thinking about how devastating it’s been for me, somebody who’s not, I only knew him a couple of years, although at quite an important time, spent a lot of time together, that kind of how damaging it is for others, I think it’s been overall a protective experience, to be honest. And so yeah, although it’s likely to have made me think about it a lot more than I would have done normally.”* (Male, 40s, bereaved by the suicide of his friend in young adulthood)

*“That I know I couldn’t do it. The mess that he left behind, the trauma that it caused, the distress, the damaged lives. No. No. Not a chance would I want to be responsible for that.”* (Female, 50s, bereaved by the suicide of her brother)

*“…when I’m not really thinking too hard about it, it kind of subconsciously is like ‘oh right, yes, suicide is a thing, that, you know, could happen, is something I could do’ and when I’m actively thinking about it or then that’s when I guess I get the ‘but it was so awful for everyone’ kind of thing. … Yeah, almost like a reminder when it gets really bad and I’m actually thinking about it, it’s almost, not that I have a pros and cons list, but if I was making a pros and cons list at that point, you know, weighing it up at, that’s when I’d be like ‘remember how bad it was for everybody’ whereas I feel like when I’m just kind of loftily saying things like ‘I don’t really care about like’, I’m like ‘oh well, suicide is always an option’.”* (Female, 20s, bereaved by the suicide of her father)

*“So, it was like constant last year, constant. And, you know, the only thing that’s stopped me because I didn’t want me kids to turn out as much of a nutter as I’ve turned out”* (Female, 40s, bereaved by the suicide of her brother and her cousin)

*“So cos you can be like right on the edge, sitting there with a [details of method redacted], or whatever, right on the edge, and then it hits you. The boys. You know. How could you, you’re gonna have to sort of just get on with this, you know. This isn’t just about you, and you start seeing the bigger picture again, you’ve got to…you need something to get you to see that bigger picture before it’s too late. I think if someone is in a situation where they’re just left, and there’s just nothing there for them to, to stop, there’s no reason to stop themselves, then you would do it, you would absolutely do it. Yeah. If I didn’t have my sons during this, I’m sure I would have just done it. So, you have to have someone or something that gives you that, more than just you, feeling, that’s not just about you, you know.”* (Female, 60s, bereaved by the suicide of her ex-partner)

“…when you’re thinking about taking your own life, you think about actually the mess that comes afterwards…Well, it’s still a mess now. it hasn’t been resolved.” (Male, 60s, bereaved by the suicide of his daughter)

- - 1. *Suicide being a permanent solution to a temporary problem*

*“It’s all very much of that moment in time, I don’t think the problems would have been, in the long-term, insolvable.”* (Male, 60s, bereaved by the suicide of his daughter)

*“I don’t think any differently on it, just desperate, desperate that they thought that was the only way out in that second, really.”* [Female, 50s, bereaved by the suicide of her son)

*“If I have bad days, I very much talk to myself ‘all this is temporary’. That’s my mindset. Regardless of how pants I might be feeling, I think, ‘you know what, half an hour, I’ll be right’. Basically. I’ll feel differently. Everything is temporary, transient. This is how everybody is. So that’s how I think, which is why I think I struggle to understand how anybody could get to that level absolute hopelessness, basically. Can’t see the way out of it.”* (Female, 40s, bereaved by the suicide of her mother)

- 1. **Conflicting views on suicide as a potential option**

See where quotes above under 1.1 and 1.2 arise from the same individuals, including:

*“…when you feel really down, you’re very aware how much worse you can make it for other people, even if you escape. And would you do that to people that you love. If you love them, then no.”* (Female, 60s, bereaved by the suicide of her ex-partner)

1. **Impact of the method of suicide on consideration of own potential methods of suicide**
   1. **Aversion to the same method**

*“I didn’t, when I was suicidal, I wasn’t gonna [use the same method]. That was the only thing that I wouldn’t do.”* (Male, 20s, bereaved by the suicide of his brother)

*“…in order to carry out suicide…you need to be able to be in the state of calmness and shut down your emotions…that I would never be able to kill myself with the way [she] did because of the level of distress it would cause me…as soon as you get any distress or panic, in my experience, you can’t do it…so, that’s why it would not be possible to use, to do that, I’ve never considered copying anything that [she] did.”* (Female, 20s, bereaved by the suicide of her sister)

*“I’m just not brave enough to do something like that…I’m not brave enough to do what he did.”* (Female, 50s, bereaved by the suicide of her son)

“… it’s dissuaded me from it. It still pops up, and I don’t know, visuals of [details of method redacted], so I think it’s definitely, yeah, it persuaded me that wouldn’t be the best way to do it. You know, again, it seems so violent and painful. You know, I would say it’s definitely put me off.” (Male, 40s, bereaved by the suicide of his friend in young adulthood)

*“I think for me, like the thought for me, I know, that to [details of method redacted] would be the most horrific way to die. I couldn’t even bear that….so that would be something, I’d never even consider that, I wouldn’t and because [redacted details of method] I think that’s what I’d do.”* (Female, 40s, bereaved by the suicide of her friend)

*“I really have thought a lot about the fact that he chose [details of method redacted]. Just how brutal that seems…There’s gotta be better ways of doing it. And I’ve thought about it quite a lot. Less painful ways.”* (Male, 40s, bereaved by the suicide of his friend in young adulthood)

*“…I had so much f****** negative images of [it]…all the imagery I’ve had of [details of method redacted] has always been my brother. I’ve always seen him do it, and I can’t put myself in that situation. I can’t see myself in that situation because it’s him…I had to be able to see myself doing it.”* (Male, 20s, bereaved by the suicide of his brother)

*“…it’s the only way that I wouldn’t have killed myself…It would have been the best way to do it, to be honest….In a weird way it kinda makes sense because, he used to rob my clothes all the time and he used to f*** me off because they were my clothes and that was my style, and that would really p*** me off. So, I think, in a weird way, that’s how he, that’s his choice, that’s how he’s done it, so I can’t do it. It was more, yeah, no, I just wouldn’t, I was like, nah. I’ve got to think of another way, I can’t do the same as him.”* (Male, 20s, bereaved by the suicide of his brother)

- 1. **A desire to protect others from the trauma of discovery**

*“…I think, probably the most I’ve thought about is the pain that he did to others as well. That’s become quite an obsession for me…how do you, how would I do it having the minimum impact on others. And I’ve thought quite elaborate plans to try and avoid what happened in that situation, should I do it.”* (Male, 40s, bereaved by the suicide of his friend in young adulthood)

*“…I was so traumatised by what had happened to [her] and the way she looked afterwards…so I thought the best way for me is to make it easy for others when they come across me. To make it so that, I just looked like I’d fallen asleep…it was about just making [it] as least traumatic to other people. That I wasn’t inconveniencing them.”* (Female, 40s, bereaved by the suicide of her adolescent daughter)

*“And it must have just been, I mean it lasted for ages, I just remember thinking, how horrific, even at the time, I remember thinking, how horrific for the emergency services to be having to do this, for two, three hours to try and get…it felt like three hours, I think it was a couple of hours before [details of method redacted]. I did think, I just, that is something I just do not want them to have to deal with. It has to be, it has to be easy for them, when they find me.”* (Female, 40s, bereaved by the suicide of her adolescent daughter)

*“No, it was [description of method used]…I don’t think I would have done it…I think I would have probably [description of own possible method]…it would be something away from home, from people finding you. I know the devastation that causes.”* (Female, 60s, bereaved by the suicide of her ex-partner)

*“I understood what it meant to do that to other people. So I think, that probably was a block in my head… I very much understood from seeing”* (Female, 20s, bereaved by the suicide of her mother)

- 1. **Concerns around being found alive and wanting to guarantee own death**

*“…I think the only kind of definitive thing it’s given is I would want to make sure I was dead. I would hate for somebody to find me and have to feel like they had to try and revive me, or like, there was some sort of paramedical care to give.”* (Female, 20s, bereaved by the suicide of her father)

*“I guess I’m scared of, I would be scared of almost not dying in the way that he nearly didn’t die. I guess I would be scared of what would have happened if he’d not passed out and [details of method redacted], you know, that would be the result I would be scared of…Cos I think, I guess… whether it’s 2 minutes or 10 minutes ultimately at the end you’re dead either way.* *It would be the not dying that would be the scary bit.”* (Female, 20s, bereaved by the suicide of her father)

*“So, it was just, to me, it was about just making as, least traumatic to other people. That I wasn’t inconveniencing them, but also making it so I wasn’t terrified in the process or worse of all, not succeeding. You know, I thought, what if I crashed a car and I’ve just broken my legs and then I’m in a wheelchair for the rest of my life. So, it had to be something successful, so it was weighing all those factors up”* (Female, 40s, bereaved by the suicide of her adolescent daughter)

“*I think it was more risk, the risk of living…it was always about, what’s gonna be end game, really, is the, what’s really, what’s gonna knock me off so that I won’t be able to come back? I even thought about putting a DNR in my pocket as well*.” (Male, 20s, bereaved by the suicide of his brother)

*“…it always had to be something, once you step out, that’s it. I didn’t want to run the risk of someone finding me half dead”* (Male, 20s, bereaved by the suicide of his brother)

1. **Experience of suicidal ideation as a means of understanding the deceased’s state of mind**

*“I think it took me to a very, very scary place that I don’t ever, ever want to be in again but in some ways, I felt like, because I kept asking ‘why?’, the universe showed me why. If that makes sense? Because they say, don’t they? Be careful what you wish for, because I kept asking this question constantly, it consumed me, I feel like I was given the answer. And that’s when I kind of went ‘woah, I get it, I get it now. I understand why’, you know?”* (Female, 40s, bereaved by the suicide of her friend)

*“I got to that point where I was suicidal…And I didn’t care that I had three children that needed their Mum and I didn’t care that I have parents that loved their daughter. I didn’t care. So that, I think for me, is when I stopped asking ‘Why?’. I think that’s when the penny dropped that ultimately, I can’t blame myself, can’t blame anyone else and for the first time ever, probably understood how [deceased’s name] was feeling and that was a big moment for me.”* (Female, 40s, bereaved by the suicide of her friend)

*“It did help. It was an answer. I got the answer that had kept me awake for months on end. I got the answer. It took me, it kind of didn’t go ‘well, this is the reason why’, it actually immersed me straight into those thoughts and feelings and I was immersed in it, so, not only did I think it, I felt it. And it was like the ultimate answer. If you know what I mean? It was like the whole package. It did, it was like a lightbulb moment, it was like ‘oh my god, this is awful, and I really don’t want to feel like this, but I’m not going to torment myself anymore with that Why question’.”* (Female, 40s, bereaved by the suicide of her friend)

*“But I used to think it might have just been a very difficult decision for him, because he would have thought of everyone else. But that was not, I know that that wouldn’t have been part of his mindset because I’ve been there myself, you just don’t, you just don’t think of anyone else whatsoever, it’s all about you.”* (Male, 20s, bereaved by the suicide of his brother)

*“…when I tried to take my own life, it became blatantly obvious to me then, how [she] must have felt. Because I felt completely hopeless, completely like nothing was going to change and I can only describe it, as mental agony…I couldn’t see another way out, so it was the only way and I thought ‘this is how [she] must have felt’…And that wasn’t very helpful because I thought, ‘why didn’t she turn to me cos I would have helped her with that’, but then again, that was quite helpful, because I thought, ‘well I didn’t turn to anyone, either’, didn’t want to burden them. So, in some ways it was a useful experience…because it gave me a really deep understanding of why people take their own life.”* (Female, 40s, bereaved by the suicide of her adolescent daughter)

*“So yeah, we’ve all been ill, really…And so I think I can understand where he was, a little bit, in that you just don’t see how things can move on.”* (Female, 50s, bereaved by the suicide of her son)

*“…it’s funny because I didn’t even have the, I also got to understand that you don’t think of the stuff that you’re leaving behind either. It’s not important. It’s just grey matter, it’s just grey blur, not grey matter. You’re just in your own world of, ‘I just can’t see a way out of this, I can’t ever envisage feeling better’.”* (Female, 50s, bereaved by the suicide of her son)

*“But in some ways…It helped in a little way, because there were times when I was so angry with her…it was almost like, before I went through it, you’ve done this to our family, you have no idea what you’ve done to us. You know, it was just, you’ve inflicted this huge pain. So almost to go there helped me come to terms with that bit of grief, which is anger. It helped me understand that actually, she couldn’t help it. And that, in itself, was helpful.”* (Female, 40s, bereaved by the suicide of her adolescent daughter)

*“…when I was contemplating killing myself, it was just, and that’s why I think as well, because of that it might have felt quite happy and normal on that morning, because when that decision, when you’re on that cusp of making that decision, I do feel serene, you know, quite happy, first time you feel happy for a long time, because to make that decision and it’s as if you’ve made, got actual some control and that’s the only control you can have over your life, is to decide that you’re not gonna do it anymore.”* (Female, 40s, bereaved by the suicide of her brother and her cousin)

*“I feel like in the immediate aftermath of my Dad’s suicide, lots of people, rightly so, felt angry or felt like it was very unfair of him and I guess probably me more than anyone else felt like I was almost standing up and saying, ‘look, you know, he wasn’t doing it, it wasn’t a selfish thing to do, he wasn’t doing it thinking about you, it wasn’t about that’ and I think, I guess I had that, I feel like, you know, it’s extra given me that perspective because I felt like, yeah, I don’t know, I feel like it’s interesting to see the aftermath and what other people think and I, you know, I feel like I can, can see that he wasn’t, you know, he wasn’t doing it, you know, he’s obviously only doing it for himself, I guess.”* (Female, 20s, bereaved by the suicide of her father)

1. **Thoughts related to reunion with the deceased**

*“It was a pull, you know that balance I’m talking about, that was one of the things on the balance that would say ‘this is a great idea’, to put it bluntly, you know, it was like, suicide’s a good option because it means that I’ll see [her] again. It was an attractive option for that….and it was one way of stopping the pain…I wasn’t just going to stop the pain. No, I was going to stop the pain with bells on with seeing [her] again.”* (Female, 40s, bereaved by the suicide of her adolescent daughter)

*“It was very attractive. It was an influence on me wanting to die by suicide”* (Female, 40s, bereaved by the suicide of her adolescent daughter)

*“I can’t do this anymore, I need to see [deceased’s name] and this would be a great way of seeing her.”* (Female, 40s, bereaved by the suicide of her adolescent daughter)

*“it’s a downward spiral because you want to be dead and you want to be with the other person and they’re like ‘do it, do it, do it, do it’. Yeah, it’s not fun.”* (Male, 20s, bereaved by suicide of his brother)

*“When I really, when I did want to die, that was about being with him quite a lot. When I was fully suicidal, a lot of it was like, was around, kind of, wanting to be with him.”* (Male, 20s, bereaved by the suicide of his brother)

*“Sometimes that’s an easy place to go to in your head, or when things are overwhelming or stuff, if you’ve had a particularly difficult day or if someone had triggered me or if I feel particularly unwell, I will go into that kind of zone of ‘I want to be with them, I just need that, they want me to come back’, that’s why it’s difficult when people say ‘oh well they’re together, or they’re watching over you, or they’re…’, cos then I just think ‘well, then I obviously want to go and be there’ and I don’t know what I think about life after death or anything like that, have no kind of firm beliefs about anything, but I can still go in my head to a place of ‘well I’ll be with them, that’ll be better, I’ll be with them, I’ll get to see them’.”* (Female, 20s, bereaved by the suicide of her sister)

*“Longing and aching can bring the feelings, but then bring a sense of knowing, a sense of comfort, really, in knowing that they’re still part of your life. Whereas, the reuniting is much more associated with suicidal ideation and yeah, the reuniting is a lot more connected to maybe the wanting to not be here”* (Female, 20s, bereaved by the suicide of her sister)
